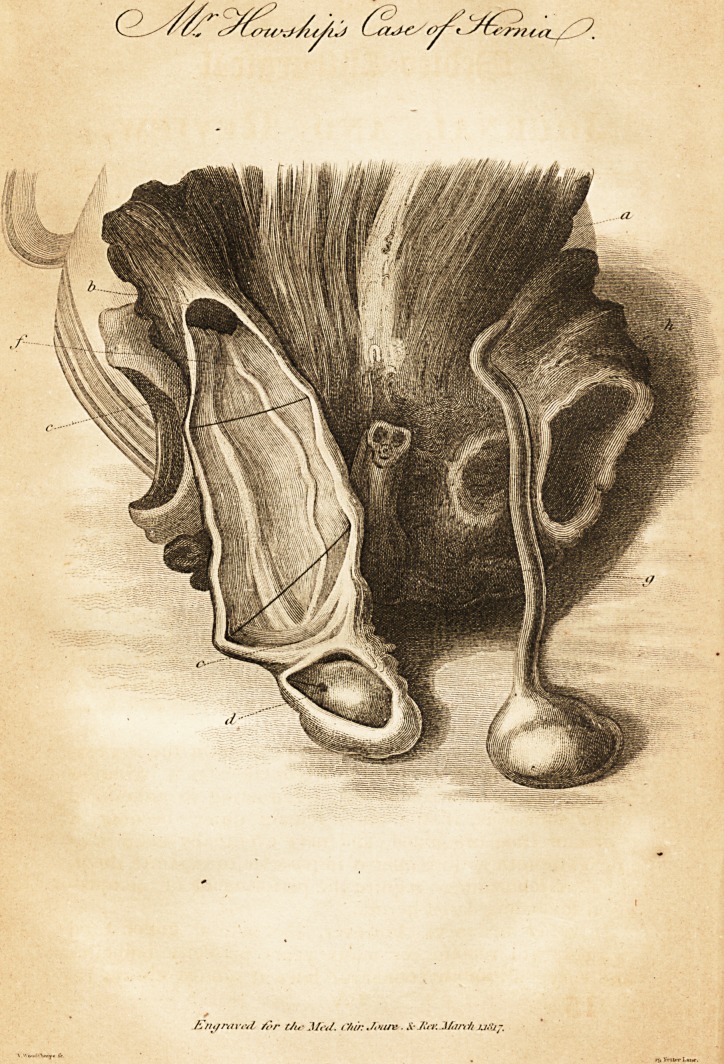# Observations on Hernia, Illustrated by a Case and Dissection

**Published:** 1817-03

**Authors:** John Howship

**Affiliations:** Surgeon


### Medico-Chirurgical Journal and Review.

VOL. III.] MARCH, 1817. [NO. 15.

### PART I.

### ORIGINAL COMMUNICATIONS.

—— quid utile ——

### *Observations on Hernia, illustrated by a Case and Dissection.*

By JOHN HOWSHIP, Surgeon.

THE consideration of the various circumstances under which some part of the contents of the abdomen may protrude, is so far interesting that, in fact, no department of surgical knowledge is more important, in a practical point of view; for hernia is in every case of consequence, and may at any moment become a very serious and most alarming complaint.

In some instances a rupture may, even in the earliest stages of its progress, become irreducible from irritation having produced inflammation, terminating in adhesions. These adhesions, if the parts remain quiet, become in course of time organized, and may eventually acquire so much strength as to render it impossible to separate them, should circumstances require the performance of the operation for strangulated hernia.

In other instances, however, the hernia, under total neglect, will remain for many years perfectly reducible and easy, although sooner or later it almost always becomes adherent and subject to all the serious accidents occasionally connected with that state. Unfortunately, when once a hernia has become adherent, and the patient is obliged to seek relief from surgery, there is no mode of determining accurately, previous to the operation being commenced, in what state the parts may be found as to the propriety, or even practicability of reduction.

The intestine, confined within the hernial sac, may suffer injury from the irritation of its contents; and on this principle, ulceration sometimes commences upon its internal membrane, extending itself outwards through the muscular and peritoneal coats of the bowel; or it may undergo the changes resulting from mortification; and while those portions of the intestinal canal passing out from the abdomen, inflame and adhere to the surrounding parts, the gangrenous portion shall ulcerate its way out through the integuments; and under the latter deplorable circumstances, in some rare instances, the case has been rendered still more complex, by a part of the intestine forming a prolapsus, with inversion of the gut.

But as various histories, in illustration of the above circumstances of disease, have been already brought forward in the "Practical Observations in Surgery," with cases and dissections, which I have just published, it is unnecessary to enlarge upon these points.

The protrusion of a portion of intestine, at the time of birth, or very soon after that period, has been usually distinguished from hernia, arising at a later date by the circumstance of the protruded bowel being found to be in contact with the testicle, from the accident having taken place previously to the closing of the aperture, by which the testicle passed down from the abdomen; in which state it has received the name of congenital hernia.

The following instance, however, shews that hernia may make its first appearance in very early infancy, without being at all connected with the tunica vaginalis testis.

A. B. a male infant, was born in perfect health; but being a strong, restless, passionate child, was observed, before he was three weeks old, to have a small swelling in the right groin, for which the child was brought to me. On examination, there was evidently a protrusion of the bowel, although it was perfectly reducible. The testicle was distinguished immediately below the intestine, but it was not easy to determine whether it was in contact with the contents of the hernial sac, or not.

As the swelling rapidly increased, bandages of various kinds were tried, but failed. A very small and light steel truss was subsequently adapted to the body of the infant, but the frequently wet state of the napkins very soon destroyed the springs, and the instrument broke. At the age of 13 months, the child died from the measles, and being allowed to examine the state of the parts, I was furnished with the opportunity for making the drawing, of which the annexed engraving is a representation.

Mill Street, Hanover Square,
December 11, 1816.

### Explanation of the Plate.

a. The anterior muscular parietes of the abdomen.
b. The opening from the abdomen forming the neck of the hernial sac.
c. The inferior extremity of the sac.
d. The testicle in its tunica vaginalis, distinct from the hernial sac; the cavity being rendered more apparent by the introduction of a bristle.
e. The spermatic vessels passing down at the posterior part of the sac, the sides of which are kept separate by two bristles.
f. The vas deferens seen passing down at some distance from the spermatic vessels.
g. The root of the penis.
h. The spermatic cord and testicle, on the left side.

## Figures and Tables

**Figure f1:**